# Association Between SLCO1B1 Gene T521C Polymorphism and Statin-Related Myopathy Risk: A Meta-Analysis of Case–Control Studies

**DOI:** 10.1097/MD.0000000000001268

**Published:** 2015-09-18

**Authors:** Qingtao Hou, Sheyu Li, Ling Li, Yun Li, Xin Sun, Haoming Tian

**Affiliations:** From the Department of Endocrinology and Metabolism, West China Hospital, Sichuan University, Chengdu, China (QH, SL, HT); Chinese Evidence-Based Medicine Center, West China Hospital, Sichuan University, Chengdu, China (LL, XS); and Department of Endocrinology and Metabolism, The Third People's Hospital of Chengdu, Chengdu, China (YL)

## Abstract

Statin-related myopathy is an important adverse effect of statin which is classically unpredictable. The evidence of association between solute carrier organic anion transporter 1B1 (SLCO1B1) gene T521C polymorphism and statin-related myopathy risk remained controversial. This study aimed to investigate this genetic association.

Databases of PubMed, EMBASE, Chinese Biomedical Literature Database (CBM), China National Knowledge Infrastructure (CNKI), Chinese Scientific Journals Database, and Wanfang Data were searched till June 17, 2015. Case-control studies investigating the association between SLCO1B1 gene T521C polymorphism and statin-related myopathy risk were included. The Newcastle–Ottawa Scale (NOS) was used for assessing the quality of included studies. Data were pooled by odds ratios (ORs) and their 95% confidence intervals (CIs).

Nine studies with 1360 cases and 3082 controls were included. Cases of statin-related myopathy were found to be significantly associated with the variant C allele (TC + CC vs TT: OR = 2.09, 95% CI = 1.27–3.43, *P* = 0.003; C vs T: OR = 2.10, 95% CI = 1.43–3.09, *P* < 0.001), especially when statin-related myopathy was defined as an elevation of creatine kinase (CK) >10 times the upper limit of normal (ULN) or rhabdomyolysis (TC + CC vs TT: OR = 3.83, 95% CI = 1.41–10.39, *P* = 0.008; C vs T: OR = 2.94, 95% CI = 1.47–5.89, *P* = 0.002). When stratified by statin type, the association was significant in individuals receiving simvastatin (TC + CC vs TT: OR = 3.09, 95% CI = 1.64–5.85, *P* = 0.001; C vs T: OR = 3.00, 95% CI = 1.38–6.49, *P* = 0.005), but not in those receiving atorvastatin (TC + CC vs TT: OR = 1.31, 95% CI = 0.74–2.30, *P* = 0.35; C vs T: OR = 1.33, 95% CI = 0.57–3.12, *P* = 0.52).

The available evidence suggests that SLCO1B1 gene T521C polymorphism is associated with an increased risk of statin-related myopathy, especially in individuals receiving simvastatin. Thus, a genetic test before initiation of statins may be meaningful for personalizing the treatment.

## Abstract 2

Supplemental Digital Content is available in the text

## INTRODUCTION

The 3-hydroxy-3-methylglutaryl coenzyme A (HMG CoA) reductase inhibitors, also known as statins, are recommended as candidate drugs for the treatment of hypercholesterolemia and prevention of cardiovascular disease. Although their clinical benefits have been undoubted, a series of potential adverse events especially myopathy have been widely reported.^1,2^ However, there has been no consensus on the definition of statin-related myopathy and the clinical spectrum has ranged from the mild myalgia or asymptomatic creatine kinase (CK) elevation to the rare but fatal rhabdomyolysis.^3,4^

The underlying pathogenesis of statin-related myopathy is still uncertain. Potential contributing risk factors include drug properties, concomitant interacting medications, individual demographic characteristics, co-morbidities, and genetic factors.^5^ Genetic factors play a crucial role in the development of statin-related myopathy. Recently, some studies have clarified the association between the polymorphisms of solute carrier organic anion transporter 1B1 (SLCO1B1) gene and statin-related myopathy risk. Among them, the T521C polymorphism has been the focus of much interest and debate.^6–8^

Although a number of studies have reported the association of SLCO1B1 gene T521C polymorphism and statin-related myopathy risk, considerable discrepancies among them have made the real relationship vague. Moreover, statin-related myopathy particularly severe myopathy or rhabdomyolysis has created great health and economic burden because of the widely use of statins in clinical practice, even though the incidence of myopathy is not very high. Thus, whether the genetic test before satin initiation in high risk individuals is cost-effective or not is very important. In order to overcome the limitations of individual studies and estimate the predictive value of genetic testing, we conducted a comprehensive and quantitative analysis to systematically assess the genetic association between T521C polymorphism and the risk of statin-related myopathy.

## METHODS

### Literature Search

The EndNote software version X7 (Thomson Reuters Corporation, Toronto, Ontario, Canada) was used throughout the searching process. The electronic databases of PubMed, EMBASE, Chinese Biomedical Literature Database (CBM), China National Knowledge Infrastructure (CNKI), Chinese Scientific Journals Database, and Wanfang Data were searched till June 17, 2015. The combination of the following keywords was used: (solute carrier organic anion transporter 1B1 or SLCO1B1 or organic anion transporter polypeptide 1B1 or OATP1B1) and (myopathy or myalgia or myositis or rhabdomyolysis or creatine kinase or CK) and (statin or rosuvastatin or fluvastatin or pravastatin or simvastatin or cerivastatin or lovastatin oratorvastatin or pitavastatin). The Medical Subject Heading (MeSH) was also used during search when available. Bibliographies of identified studies were searched to make sure all the potentially relevant studies included. We also contacted with the author by Email for sufficient data. The language was limited to either English or Chinese. No ethical approval and patient consent are required because all analyses were based on previous published studies.

### Inclusion and Exclusion Criteria

Studies were included if they met the following criteria: the case–control design investigated the association between SLCO1B1gene T521C polymorphism and the risk of statin-related myopathy; the study provided enough information to calculate the gene frequencies in both case and control group; myopathy was defined according to CK elevations or muscle symptoms or both.

### Study Selection

Two reviewers (QH and SL) independently screened the titles and abstracts, and then the full texts for eligibility. Discrepancies between the 2 reviewers’ selections were resolved by consensus.

### Data Extraction and Quality Assessment

The following information was extracted from each study by 2 reviewers (QH and SL) independently: first author, year of publication, country, ethnicity, genotyping method, case definition, control source, age, gender, baseline matching, statin type, sample size (case/control), genotype, and/or allele frequencies in cases and controls. The Newcastle–Ottawa Scale (NOS) was used for assessing the quality of included studies.^9,10^ Any disagreements were resolved through discussion.

### Statistical Analysis

The STATA software version 12.0 (STATA Corporation, College Station, TX) was used throughout the analyses. Between-study heterogeneity was estimated using the chi-squared test and quantified with the I^2^ statistic, and *P* < 0.10 was considered the presence of statistical heterogeneity.^11^ The odds ratios (ORs) with their 95% confidence intervals (CIs) were estimated on the basis of a dominant model (TC + CC vs TT) and an allelic model (C vs T). The pooled ORs were determined by the Z-test and a *P* value below 0.05 was considered statistically significant. Subgroup analyses by statin type, control source, and age were also conducted to explore the sources of heterogeneity. Sensitivity analysis was carried out to determine the robustness. Begg's test and Egger's test were employed to evaluate the publication bias.^12^

## RESULTS

### Study Characteristics

**FIGURE 1:**
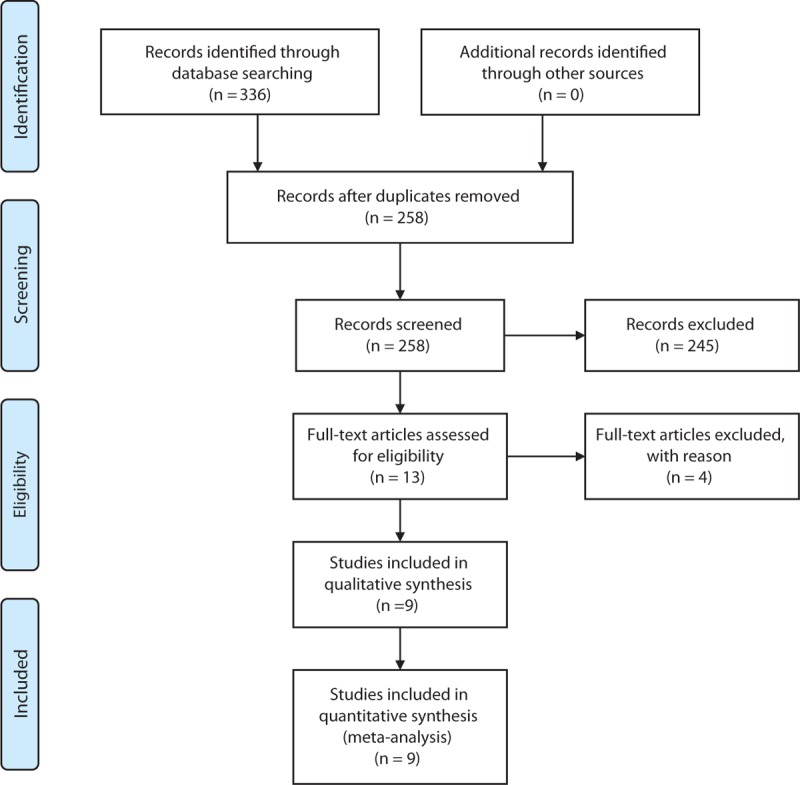
Flow diagram for study identification and inclusion.

A total of 336 articles were originally identified. After excluding 78 duplicate articles, 258 articles were left for abstract screening and full-text assessment. After abstract screening, 13 studies^13–25^ were left for full-text assessment. After evaluating the full-text according to inclusion and exclusion criteria, 9 studies^14–22^ including 1360 cases and 3082 controls were eligible for further meta-analyses (Figure 1). The baseline characteristics of studies included are displayed in Table 1. Genotype and allele frequencies are shown in Table 2. The definitions of statin-related myopathy were based on elevations of serum CK level with or without muscular symptoms except for 1 study.^18^ In which patients with self-reported myalgia were classified as cases. Severe statin-related myopathy was defined as CK > 10× the upper limit of normal (ULN) or rhabdomyolysis.^26^ There were 4^14,15,17,19^ studies performing on simvastatin, 4^15,17,19,22^ on atorvastatin, and 4^14,15,17,20^ on severe myopathy. Seven studies^14–18,20,21^ examined Caucasian subjects, whereas 2 studies^19,22^ examined mixed Caucasians. One study^22^ reported the association of SLCO1B1 genotypes with myalgia and CK elevation separately from the same population group, so we just extracted the data based on CK elevation, because most existing definitions of statin-related myopathy were based on biochemical standards rather than clinical phenotypes.^27^ Valid genotyping methods were applied throughout all the studies. The distribution of genotypes in the control group deviated from Hardy–Weinberg equilibrium (HWE) in 1 study^15^ with a *P* value 0.04.

**TABLE 1:**
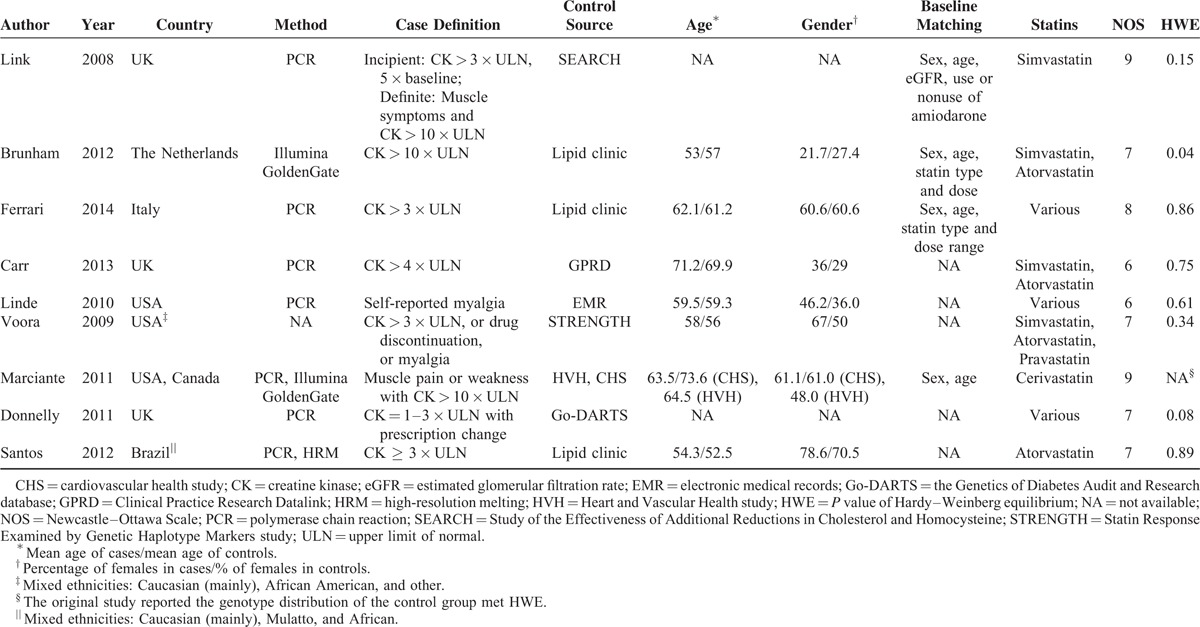
Baseline Characteristics of Studies Included

**TABLE 2:**
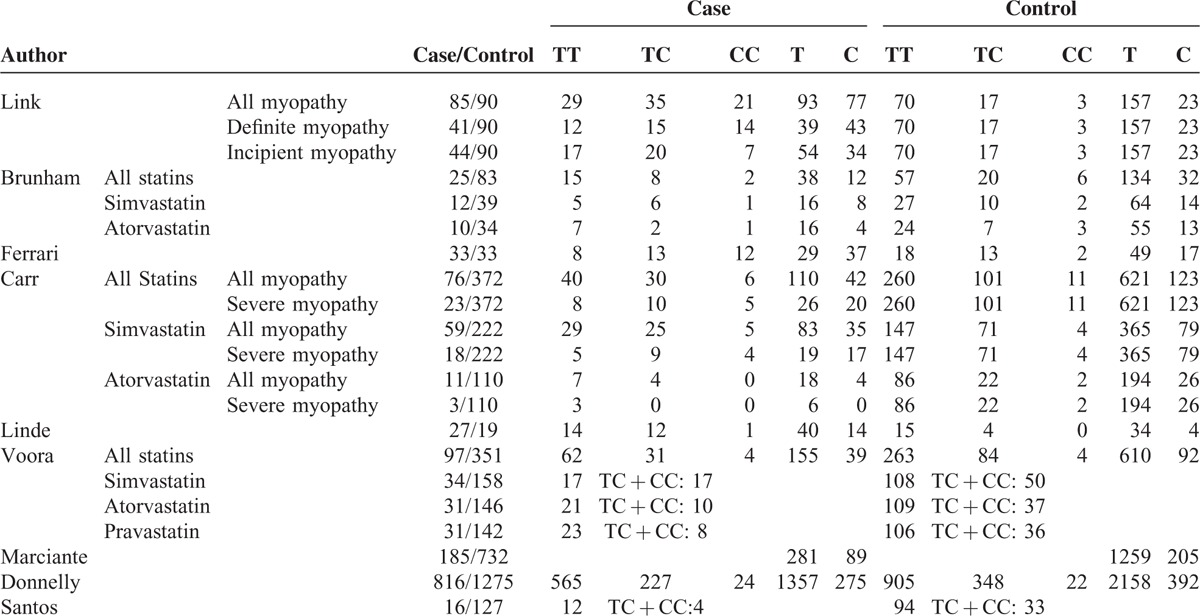
Genotype and Allele Frequencies of Studies Included

### Quality Assessment

The NOS was adopted to evaluate the methodological quality of all included studies. All studies showed a relatively high quality with a mean score of 7, ranging from 6 to 9 (Table 1). Four studies^14–16,20^ used matched controls while the other 5 studies^17–19,21,22^ did not provide available data on baseline matching. Three studies^14,19,20^ genotyped participants from previous clinical trials, while 6^15–18,21,22^ recruited patients from the real world. One study^21^ was carried out in patients with type 2 diabetes.

### Meta-Analysis

**FIGURE 2:**
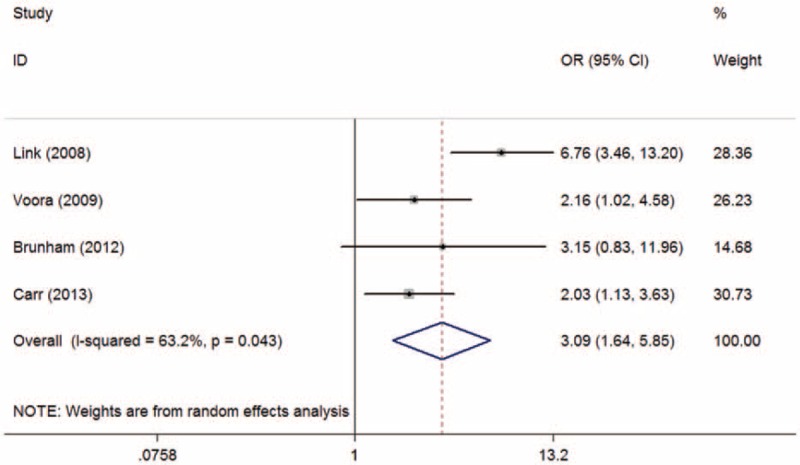
Meta-analysis of simvastatin-related myopathy risk and SLCO1B1 gene T521C polymorphism based on dominant model (TC + CC vs TT). CI = confidence interval; OR = odds ratio.

**FIGURE 3:**
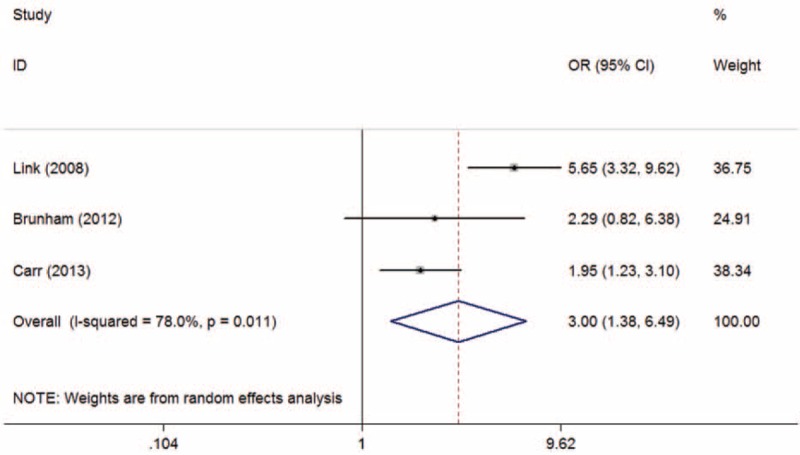
Meta-analysis of simvastatin-related myopathy risk and SLCO1B1 gene T521C polymorphism based on allelic model (C vs T). CI = confidence interval; OR = odds ratio.

**FIGURE 4:**
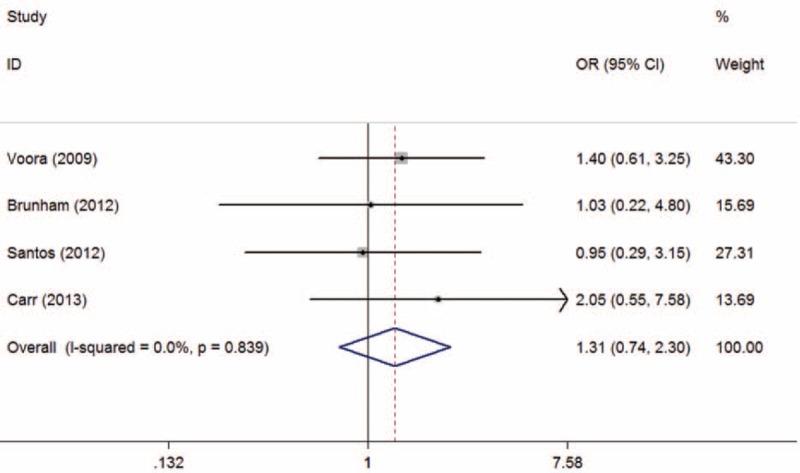
Meta-analysis of atorvastatin-related myopathy risk and SLCO1B1 gene T521C polymorphism based on dominant model (TC + CC vs TT). CI = confidence interval; OR = odds ratio.

**FIGURE 5:**
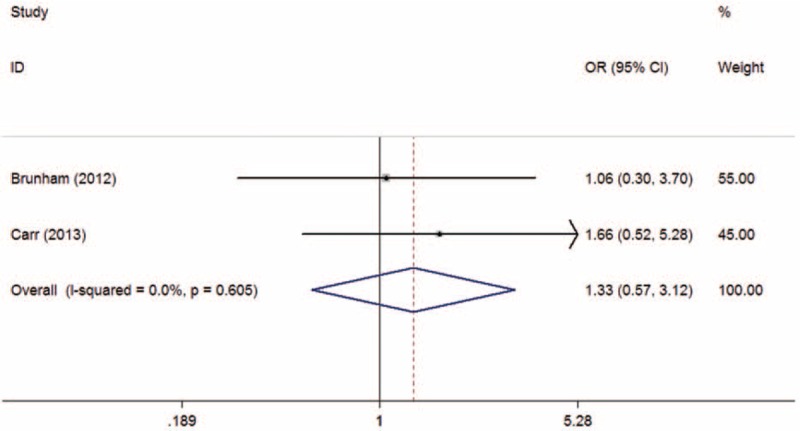
Meta-analysis of atorvastatin-related myopathy risk and SLCO1B1 gene T521C polymorphism based on allelic model (C vs T). CI = confidence interval; OR = odds ratio.

The overall and subgroup analyses are shown in Table 3. A statistically significant association between SLCO1B1 gene T521C polymorphism and statin-related myopathy risk was found (TC + CC vs TT: OR = 2.09, 95% CI = 1.27–3.43, *P* = 0.003; C vs T: OR = 2.10, 95% CI = 1.43–3.09, *P* < 0.001), indicating that the allele C carriers may be more intolerant to satins. The association was more obvious when statin-related myopathy was defined as CK > 10 × ULN or rhabdomyolysis (TC + CC vs TT: OR = 3.83, 95% CI = 1.41–10.39, *P* = 0.008; C vs T: OR = 2.94, 95% CI = 1.47–5.89, *P* = 0.002). However, the results were inconsistent when stratified by statin type. The association was statistically significant in individuals receiving simvastatin (TC + CC vs TT: OR = 3.09, 95% CI = 1.64–5.85, *P* = 0.001; C vs T: OR = 3.00, 95% CI = 1.38–6.49, *P* = 0.005) (Figures 2 and 3), but not in those receiving atorvastatin (TC + CC vs TT: OR = 1.31, 95% CI = 0.74–2.30, *P* = 0.35; C vs T: OR = 1.33, 95% CI = 0.57–3.12, *P* = 0.52) (Figures 4 and 5).

**TABLE 3:**
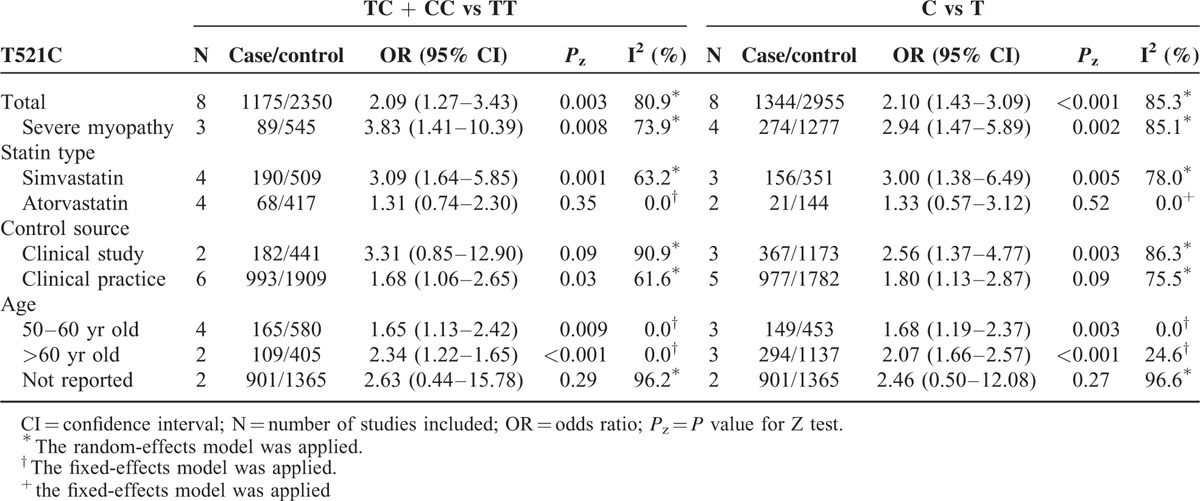
Main Results of Meta-analyses based on Dominant and Allelic Models

Subgroup analyses based on control source showed that the association in clinical practice (TC + CC vs TT: OR = 1.68, 95% CI = 1.06–2.65, *P* = 0.03; C vs T: OR = 1.80, 95% CI = 1.13–2.87, *P* = 0.01) was more robust than that in clinical study (TC + CC vs TT: OR = 3.31, 95% CI = 0.85–12.90, *P* = 0.09; C vs T: OR = 2.56, 95% CI = 1.37–4.77, *P* = 0.003) and the relationship in >60 years old age group (TC + CC vs TT: OR = 2.34, 95% CI = 1.22–1.65, *P* < 0.001; C vs T: OR = 2.07, 95% CI = 1.66–2.57, *P* < 0.001) was stronger compared with that in 50-60 years old age group (TC + CC vs TT: OR = 1.65, 95% CI = 1.13–2.42, *P* = 0.009; C vs T: OR = 1.68, 95% CI = 1.19–2.37, *P* = 0.003) when stratified by age (Table 3).

### Publication Bias and Sensitivity Analysis

Publication bias was not detected in Begg's and Egger's tests for both contrast models (all *P* > 0.05) (Table 4). The results remained statistically significant by excluding 1 study at a time based on a sensitivity analysis (see Figures S1 and S2, http://links.lww.com/MD/A405, which illustrate the results of sensitivity analyses).

**TABLE 4:**
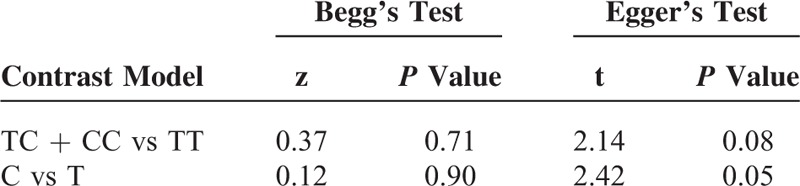
Results of Publication Bias From Begg's and Egger's Tests

## DISCUSSION

In summary, we have demonstrated that SLCO1B1 gene T521C polymorphism is significantly associated with statin-related myopathy, the variant C allele may increase the risk of developing statin-related myopathy (especially severe myopathy). Nonetheless, when stratified by stain type, the association is significant in patients receiving simvastatin but not in patients receiving atorvastatin, which suggests that the association may be a statin type-specific. Subgroup analyses based on control source and age have revealed that the association in clinical practice and older age group was more statistically significant.

The SLCO1B1 gene is localized to an SLCO1 gene cluster in the short arm of chromosome 12 and encodes the OATP1B1 on the basolateral membrane of hepatocytes. The OATP1B1 is an influx transporter responsible for hepatic uptake of statins. The T521C polymorphism, rs4149056, is a relatively common nonsynonymous single-nucleotide polymorphism located in exon 5 of SLCO1B1 gene.^28,29^ The functional T→C transition results in a Val→Ala substitution. The substitute alanine is associated with a decreased transport function and an elevated plasma statin concentration, and consequently produces a stronger susceptibility to myopathy.^28,30^ Compared with the normal function of the homozygous wild TT genotype, the heterozygous TC genotype and homozygous mutant CC genotype has an intermediate and a low function, respectively.^31^

Although OATP1B1 is the primary transporter for the uptake of statins, organic anion transporter polypeptide 1B3 (OATP1B3) and organic anion transporter polypeptide 2B1 (OATP2B1) also take part in the process of some specific statins. Our data indicated that T521C polymorphism was mainly associated with simvastatin-related myopathy rather than atorvastatin-related myopathy. The underlying mechanism may be partly explained by varying degrees of contributions of other organic anion transporter polypeptides (OATPs) to the hepatic uptake that simvastatin is mainly uptaken by OATP1B1, whereas atorvastatin is a substrate of both OATP1B1 and OATP2B1.^32^ Meanwhile, pharmacokinetic studies have demonstrated that the area under the plasma concentration-time curve of active simvastatin acid and atorvastatin is 221% and 144% greater in 521CC genotype than that in 521TT genotype, respectively.^33,34^

These findings of subgroup analyses based on control source and age can be explained by the following reasons. The incidence of statin-related myopathy in clinical practice is higher than that in clinical studies, because comorbidities and polypharmacy are generally excluded in clinical studies, and moreover, the uncertainty of a precise recording of all adverse events due to the study design in clinical studies may underestimate the association.^35^ Senior age itself is a risk factor of statin-related myopathy, and it may be explained by some age-dependent physiologies (such as declines in renal and hepatic function) and age-related risk factors for myopathy (such as multisystem diseases and concomitant medications). Thus, we should be more cautious when prescribing statins to the elder patients in clinical practice.^4^

Our findings are consistent with the previous results reported by Carr et al.^17^ Moreover, our study is the first one that systematically assessed the association between SLCO1B1 gene T521C polymorphism and statin-related myopathy risk in detail based on a large sample size and different gene contrast models both in the whole population and various subgroups. Other strengths of this study include the relatively high quality of included studies evaluated by the NOS and the robust results verified by the sensitivity analyses. More importantly, the results of our study may serve as evidence for guiding the preemptive SLCO1B1 testing by identifying individuals with high risk of simvastatin-related myopathy and thereby optimizing the statin therapy to avoid drug-related myopathy and promote a stronger adherence. Recently, the Clinical Pharmacogenomics Implementation Consortium Guideline recommended a lower dose of simvastatin or an alternative statin as well as the potential utility of routine CK surveillance for patients with a reduced-function C allele.^31^ Apart from the statin-related myopathy, the T521C polymorphism was also reported to be potentially associated with the cholesterol-lowing effect.^14,21^ Thus, a preemptive genetic test may not only minimize the risk but also maximize the efficacy of statin therapy and therefore get a better risk–benefit ratio.

Evidence of publication bias was not detected in both the dominant and allelic models in Begg's and Egger's tests. However, in some ways, the publication bias was unavoidable because of the limited databases searched and only English and Chinese publications included.

There are also several limitations. Firstly, the definitions of statin-related myopathy are various which may be a source of heterogeneity in our study. However, there has been little concensus on the definition of statin-related myopathy since the concept was put forward,^4,27,36^ which has made it difficult to estimate the data based on different definitions. Secondly, subgroup analyses based on statin doses and other statin types were unavailable due to insufficient data. Recently, Hubacek et al found there was no association between SLCO1B1 gene rs4363657 (T89595C) polymorphism and the risk of myalgia/myopathy in Czech patients treated with low statin doses (simvastatin or atorvastatin, 10 or 20 mg per day).^23^ Therefore, our results should be interpreted with caution, especially high dosage is a proved risk factor of statin-related myopathy.^4,5^ Further research is required to confirm the dose-dependent and type-specific risk of statin-related myopathy associated with different candidate SLCO1B1 genes, including rs4149056 (T521C), rs2306283 (A388G),^21,22,24,37^ rs4363657 (T89595C),^14,23^ and rs11045818 (G411A),^37^ which potentially influence the pharmacokinetics of some specific statins. Thirdly, the baseline characteristics between cases and controls were not well-matched in all the included studies. Now that the development of statin-related myopathy is multifactorial, the potential confounding factors may influence the reliability of our results. More research is necessary to further elucidate on how other genes and nongenetic factors are involved in the pathogenesis of statin-related myopathy. Finally, the ethnicity of our study was mainly focused on the Caucasian, so we may not generalize the results to other ethnicities because the variant C allele frequency differs markedly between populations.^24,25,38^ A study on different ethnicities in Brazil revealed that the frequencies of the 521C allele were highest in Amerindians (28.3%) and lowest in African descent subjects (5.7%) compared with Mulatto (14.9%) and Caucasian descent (14.8%).^39^

In conclusion, we found a significant association between SLCO1B1 gene T521C polymorphism and statin-related myopathy risk, especially in patients taking simvastatin. The variant C allele may be a strong risk factor of simvastatin-related myopathy. In clinical practice, a genetic testing might help to personalize the prescription of statins and avoid drug-related myopathy. However, genetic contribution and cost-effective analysis based on specific population should be further evaluated before further recommendation.
